# A chimeric rather than native secretion signal elicits stress-related transcriptional adaption during 75k γ-secalin production in *Komagataella phaffii*

**DOI:** 10.1093/femsmc/xtag046

**Published:** 2026-10-07

**Authors:** Kai Büchner, Thorsten Schmidt, Roland Kerpes, Thomas Becker

**Affiliations:** TUM School of Life Sciences, Chair of Brewing and Beverage Technology, Technical University of Munich, 85354 Freising, Germany; Department of Bioengineering (Sciences), Professorship Applied Informatics for Life, University of Applied Sciences Weihenstephan-Triesdorf, 85354 Freising, Germany; TUM School of Life Sciences, Chair of Brewing and Beverage Technology, Technical University of Munich, 85354 Freising, Germany; TUM School of Life Sciences, Chair of Brewing and Beverage Technology, Technical University of Munich, 85354 Freising, Germany

**Keywords:** *Komagataella phaffii*, transcriptomics, secretion signal peptides, *Secale cereale*, gluten, prolamin

## Abstract

*Komagataella phaffii* (formerly *Pichia pastoris*) is a popular host for protein production, enabling their targeted application and in-depth study. Among relevant targets are gluten proteins, which are essential for cereal-based food properties, but can also trigger immune reactions such as coeliac disease. Previously, production of rye (*Secale cereale*) prolamin 75k γ-secalin in K. phaffii was shown to yield the highest titres with the native secretion signal compared to co- and post-translational signals commonly used for enhanced production. Here, we compared the host’s transcriptomic response to 75k γ-secalin expressed with the native signal (gSec) or a chimeric OST1-pre-MATα-pro leader (S4gSec) against a non-producing null mutant. Production of the native 75k γ-secalin showed minimal impact; whereas stress-, amino acid transport-, and flocculation-related pathways were upregulated in *K. phaffii* S4gSec. Transcriptome investigation yielded *GCN4* as potential marker for transcriptomic stress monitoring during heterologous protein production. Despite *K. phaffii*’s significance as protein production host, many of its genes are only putatively annotated. Of the 31 (gSec) and 349 (S4gSec) differentially expressed genes, 29% and 59.3% are putative, respectively. This study presents a tentative *in silico* characterization of these genes, highlighting the importance of continued functional characterization of the *K. phaffii* genome.

## Teaser

Rye prolamin producing *Komagataella phaffii* shows a stress reaction to the target protein only when the its native signal peptide is replaced, implying limitations of signal sequence optimisation strategies.

## Background

*Komagataella phaffii* is a non-conventional, methylotrophic yeast widely used in the production of heterologous proteins. Its popularity stems from its ability to grow to high cell densities, a high protein secretion rate, a low tendency to hyper-manosylate proteins (Ata et al. 2021), and a constitutive base level activity of the unfolded protein response (UPR) (Guerfal et al. 2010). The UPR affects the early secretory pathway, heightening its overall capacity and thereby rendering the cells more efficient in protein production (Raschmanová et al. 2021).

*Komagataella phaffii*’s importance as an industrial production platform led to several studies of its transcriptome (Love et al. 2016, Matthews et al. 2018, Zhang et al. 2020, Yu et al. 2022). Such studies deepened the understanding of *K. phaffii*’s reaction to environmental insults and highlighted differences between *K. phaffii* and the standard yeast model *Saccharomyces cerevisiae* (Graf et al. 2008). They also detailed the organism’s reaction to the production of heterologous proteins (Hesketh et al. 2013), yielding valuable data for further optimization of the production host. General optimization strategies employ co- or post-translational import modes of nascent proteins into the endoplasmic reticulum (ER) through different signal leader sequences (Fitzgerald and Glick 2014, Barrero et al. 2018), engineering of the target protein’s N-glycosylation (Yu et al. 2017), or co-expression of chaperone proteins (Wang et al. 2023). Post-translational ER Import can be achieved through the bipartite *S. cerevisiae* Mating Factor α (MATα) secretion signal. It consists of an N-terminal pre-region that facilitates post-translational ER entry (Stern et al. 2007), and a subsequent pro-region that supports ER import by the BiP chaperone through a molecular ratchet mechanism (Matlack et al. 1999). Other signal-sequences, such as *OST1*-pre, facilitate co-translational ER-import that results in efficient translocation during protein translation (Fitzgerald and Glick 2014, Barrero et al. 2018). While secretion signal peptides on their own were shown to aggregate almost regardless of the aggregation propensity of their cargo protein (Horgan et al. 2025), co-translational import was shown to minimize the proteins’ exposure to the cytosol and is therefore especially advantageous for aggregation-prone proteins (Voorhees and Hegde 2016). One such protein family with pronounced aggregation behaviour in native (Tosi 2012) and heterologous hosts (Rosenberg et al. 1993) is gluten, the storage proteins of Poaceae grains such as wheat, rye, and barley (Lexhaller et al. 2019). Gluten proteins are integral to the quality of grain-based foods (Day et al. 2006), yet also elicit immune system responses in susceptible individuals. One such disorder, coeliac disease, is prevalent in 0.7%–1.4% of the global population (Singh et al. 2018). This highlights the further need for medical and food science research into these seed storage proteins, which can be aided by the efficient production of single gluten protein family members. A preceding study showed *K. phaffii* as a suitable production host for rye prolamin 75k γ-secalin, while optimization strategies led to adverse results: replacing the native secretion signal with the post-translational *OST1*-pre signal diminished the protein yield and the introduction of the *OST1*-pre-MATα-pro eliminated all target protein detectable by immunoblotting (Büchner et al. 2025). Here, deep sequencing was used to elucidate transcriptome differences between the non-producing control strain, strains producing secalin with either its native signal sequence, or the hybrid *OST*1-pre-MATα-pro secretion leader.

## Materials and methods

### Strains

All strains used in this study are based on *K. phaffii* BSY BG11 (mut^S^ phenotype) and plasmids obtained from Bisy GmbH, Austria. Strain construction was described previously (Büchner et al. 2025, Kastberg et al. 2025). In short, *K. phaffii* BSY BG11 wild type cells were transformed by electro-transformation with the rye *(Secale cereale* cv. Elias*) Sec2* gene in the vector BSY3Z (BSY GmbH, Austria), constructed and linearized through overlap extension PCR (Büchner et al. 2025). The original Zeocin™ resistance of the mutant strain *K. phaffii* BSY3Z-gSec was replaced by electro-transformation of linear cassettes harbouring native rye *Sec2*, or its mutant variants rye *Sec2* [1_79del; 92delG] and *OST1*-pre-MATα-pro rye *Sec2*, and a resistance gene against the aminoglycosyde antibiotic G418. The correct replacement of the initial vector was confirmed by replica plating on YPD plates containing Zeocin™ and G418, respectively. *Komagataella phaffii* gSec produces the prolamin 75k γ-secalin under the control of the methanol inducible promoter P_CAT1_. In the strain *K. phaffii* S4gSec, the native 75k γ-secalin secretion signal was replaced by the signal-sequence chimera *OST1*-pre-MATα-pro. The truncated 75k γ-secalin gene was used to replace the native gene in *K. phaffii* gSec to create *K. phaffii* ΔgSec, a null mutant that does not produce the target protein but still harbours the expression cassette. The integration loci of the mutation cassettes were determined by long read sequencing of the whole *K. phaffii* genome as described below.

### Media and cultivation

Working cultures were stored at 4°C on standard yeast peptone dextrose (YPD) agar plates (10 g/l yeast extract, 20 g/l peptone, and 20 g/l glucose, and 20 g/l agar), supplemented with 1 mg/ml G418-sulfate (Geneticin™, Thermo Fisher Scientific Inc., USA). Cultivations were carried out in baffled shaker flasks filled to 1/10 of their nominal volume with medium, incubated at 28°C and 180 rpm. For each strain, four independent pre-cultures were grown overnight in BMG1 (1.34% (w/v) YNB, 1% (w/v) glucose, 4×10 − 5% (w/v) biotin, 200 mM potassium phosphate buffer pH 6.0) from single colonies. The cells were spun down and used to inoculate BMG1 medium to an optical density (OD_600_) of 0.5 for 48 h glucose batch cultivations. For P_CAT1_ induction, cells were inoculated in BMM1 (1.34% (w/v) YNB, 0.5% (v/v) methanol, 4 × 10− 5% (w/v) biotin, 200 mM potassium phosphate buffer pH 6.0) with a start-OD_600_=10 ± 0.4 and cultivated for 96 h, while the medium was supplemented every twelve hours with 0.5% of the total start volume in methanol. Sterile water was added to compensate losses through evaporation. The OD_600_ was measured at every feeding time-point and differences between strains were tested for significance with a two-tailed t-test for independent groups assuming equal population variances.

### Protein extraction, immunoblotting, and competitive gliadin ELISA

*Komagataella phaffii* cells were lysed mechanically with Ø0.5 mm glass beads in lysis buffer (0.137 M NaCl, 1 mM PMSF, 0.1 M EDTA), the pellets were then extracted for 60 min at 60°C with 55% (v/v) propan-2-ol, 45% (v/v) lysis buffer, supplemented with 6% (w/v) DTT (Kanerva et al. 2008). Aliquots corresponding to 5 × 10^7^ cells were then run on a hand-cast 10% Tris-Glycine SDS-PAGE gel, blotted onto a nitrocellulose membrane and incubated overnight at 8°C with a 1:1.000 diluted anti-gliadin antibody coupled to horseradish peroxidase (HRP) (A1052, Sigma-Aldrich Inc., USA). Developer solution (TBS with 0.3 mg/ml 4-Chloro-1-Naphtol in methanol and 0.3% (v/v) H_2_O_2_) was applied at room temperature until bands became visible.

RIDASCREEN® Gliadin competitive ELISA (R-Biopharm AG, Germany) was used to determine 75k γ-secalin concentrations in extract aliquots corresponding to 2 × 10^6^ cells. The manufacturer’s protocol was followed with exception of the extraction step, which was omitted. Plate reads were processed with RIDASOFT® Win.NET Food & Feed (Art. Nr. Z9996FF, R-Biopharm AG, Germany).

### Isolation of genomic DNA, library preparation, and long read sequencing

High molecular weight (HMW) DNA was extracted from cell pellets according to (Heimhilcher et al. 2025). A pooled, sequencing-ready library was created by combining one individually barcoded library per strain, prepared from the HMW DNA using the Rapid Barcoding Kit 24 V14 (SQK-RBK114.24, Oxford Nanopore Technologies, United Kingdom) according to the manufacturers instructions. The library was sequenced for 56 h on an R10.4.1 (FLO-MIN114) flow cell with a total of 1315 pores on a GridION Mk1 (Oxford Nanopore Technologies, United Kingdom). The run yielded 2.85 M reads of which 2.23 M had a min. quality score of 10. Bases were called with Dorado v2.0.0 (Oxford Nanopore Technologies 2026) using the super accuracy basecalling model dna_r10.4.1_e8.2_400bps_sup@v5.2.0 in simplex mode.

### Long read sequencing data analysis

All analyses were carried out on the public server at usegalaxy.eu (The Galaxy Community et al. 2024). The quality of the long reads was assessed with NanoPlot (Galaxy Version 1.47.0+galaxy0) (De Coster and Rademakers 2023). Reads from all sequencing runs were mapped against a *K. phaffii* reference genome (Sturmberger et al. 2016), which was concatenated with the respective vector sequences, using minimap2 (Galaxy Version 2.31+galaxy0) (Li 2018) with the map-ont profile and including query sequences for secondary alignments. The alignments were sorted by coordinates in samtools sort (Galaxy Version 2.0.8) (Li et al. 2009). Structural variants were detected with sniffles2 (Galaxy Version 2.5.2+galaxy0) (Smolka et al. 2024), with a –minsupport of 2 and without active mosaic mode, against the expanded reference genomes used in minimap2. Independent breakend (BND) events were inspected in the Integrative Genomics Viewer (IGV 2.19.2) (Robinson et al. 2011).

### RNA isolation, library preparation, and short read sequencing for transcriptomics

Four shaker flasks of each *K. phaffii* strain were harvested as described before (Büchner et al. 2025), pelleted and concentrated to an OD_600_=50 in PBS (0.8% (w/v) NaCl, 0.02% (w/v) KCl, 0.144% (w/v) Na_2_HPO_4_, 0.0245% (w/v) KH_2_PO_4_, pH 7.4) with 2% (v/v) ß-mercaptoethanol. After lysis (glass beads Ø0.5 mm, 5 min at 30 Hz) with a Qiagen TissueLyser II (Qiagen N.V., Netherlands), total RNA was extracted using the Roboklon Universal RNA Kit according to the manufacturer’s instructions (Roboklon GmbH, Germany) and RNA quality was assessed with the Bioanalyzer 2100 system (Agilent Technologies, Inc., USA). A library of four biological replicates per *K. phaffii* strain was constructed using the Illumina stranded mRNA Prep kit and sequenced on an Illumina platform NextSeq 1000 with P2 XLEAP-SBS Reagents (Illumina, USA) as dark cycle sequencing, generating 99 bp paired-end reads.

### Short read sequencing data analysis and *in silico* gene prediction

Read quality was assessed with Falco 1.2.4 (Sena Brandine de and Smith 2021) and MultiQC 1.27 (Ewels et al. 2016). The reads were then mapped against a *K. phaffii* reference genome (Sturmberger et al. 2016, Schusterbauer et al. 2025) and quantified using salmon 1.10.1 (Patro et al. 2017). Differential gene expression was analyzed in DESeq2 (Galaxy Version 2.11.40.8+galaxy0) (Love MI et al. 2014). Significance of the results was assessed with *P*-values attained by the Wald test, corrected for multiple testing with the Benjamini and Hochberg method as implemented in DESeq2. Differentially expressed genes in both datasets (*K. phaffii* gSec vs. ΔgSec and S4gSec vs. ΔgSec) were filtered for adjusted *P*-values ≤ 0.05; the *K. phaffii* S4gSec vs. ΔgSec dataset was additionally filtered by log_2_ fold change (log2(FC)) >|0.58|, as described in (Graf et al. 2008). This threshold was not applied to the *K. phaffii* gSec vs. ΔgSec dataset due to all significantly differentially expressed genes showing (log2(FC)) <|0.58|. Non-mRNA entries in all datasets, such as ribosomal and transport RNAs, internally transcribed spacers, and mitochondrial mRNA, were removed manually. Known genes were analyzed using the Gene ontology (GO) term enrichment analysis with PANTHER 19.0 (Thomas et al. 2022) with false discovery rate (FDR), and genes unmapped in this analysis were used as queries in BLASTP searches within the Methylotroph Gene Order Browser (Douglass et al. 2019). Primary protein structures corresponding to the putative genes that were differentially expressed were analyzed using InterPro (Blum et al. 2025); homologies to the *S. cerevisiae* S288C genome and proteome were investigated using BLASTP using default parameters, implemented in the NCBI database (NCBI Resource Coordinators et al. 2018).

## Results

Previously published research regarding the optimization of 75k γ-secalin production in *K. phaffii* showed a significant impact of the secretion leader signal on the protein yield (Büchner K et al. 2025). This study aims at elucidating the underlying mechanisms on a transcriptional level to provide starting points for future optimization approaches and their monitoring. In order to track any phenotypic effects of the 75k γ-secalin expression, cell growth was measured during the 96 h methanol fed-batch phase in the shaker flasks. No significant differences were detected in the development of the optical density OD_600_ (*K. phaffii* gSec vs. ΔgSec *P* = 0.34; S4gSec vs. ΔgSec *P* = 0.99; Fig. 1).

**Figure 1:**
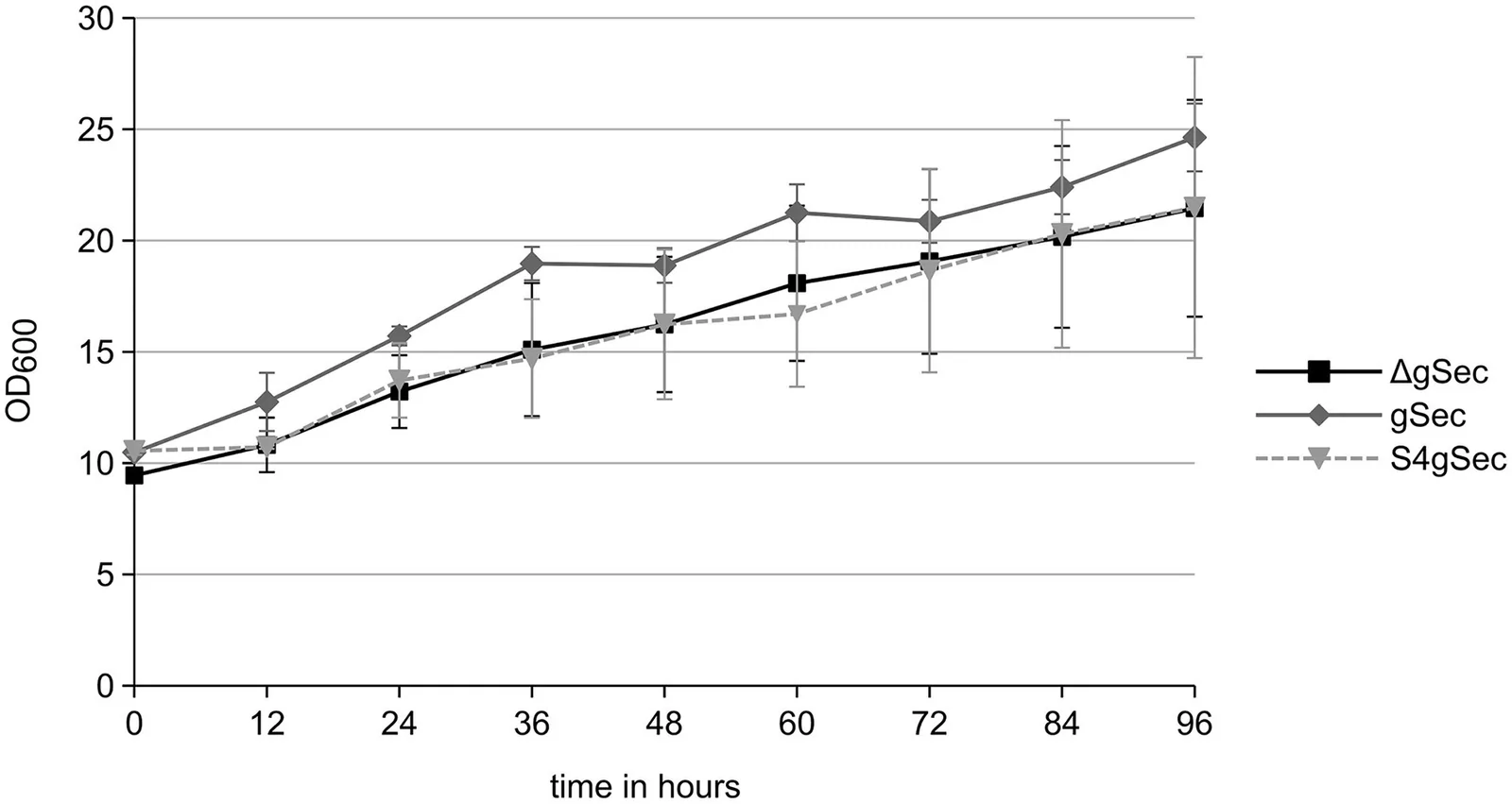
Optical density at λ = 600 nm (OD600) of *K. phaffii* shaker flask cultures (n = 4) during the methanol fed-batch phase. Error bars represent the standard deviation of the measured values.

The anti-gliadin immunoblot revealed bands only for the whole cell extract of strain *K. phaffii* gSec (Fig. 2). While the column shows less bands than reported earlier (Büchner et al. 2025), a strong band at 72 kDa implies 75k γ-secalin production. Extracts of *K. phaffii* ΔgSec and, more intriguingly, *K. phaffii* S4gSec show no bands in the blot, thereby confirming earlier results (Büchner et al. 2025). Also in accordance with these previously published results, neither immunoblot nor ELISA detected any 75k γ-secalin in the culture supernatant. ELISA measurements of the *K. phaffii* ΔgSec whole cell extracts (Fig. 3), when corrected for dilution, showed a concentration of 0.90 ± 0.27 µg 75k γ-secalin/5 × 10^7^ cells. For S4gSec, the extract contained 4.49 ± 1.05 µg 75k γ-secalin/5 × 10^7^ cells and for the native producer *K. phaffii* gSec 25.98 ± 5.84 µg 75k γ-secalin/5 × 10^7^ cells. Both yields are significantly higher than that of the null mutant (p_S4gSec_ = 7.7 × 10^−5^ and p_gSec_ = 0.018), similar to results published before (Büchner et al. 2025). With the approximation of 1 OD_600_ = 5 × 10^7^ cells/ml, the 75k γ-secalin yield of *K. phaffii* gSec amounts to 639.97 ± 155.93 mg/l at an OD_600_ of 24.63 ± 2.32 at the 96 h harvest time point.

**Figure 2:**
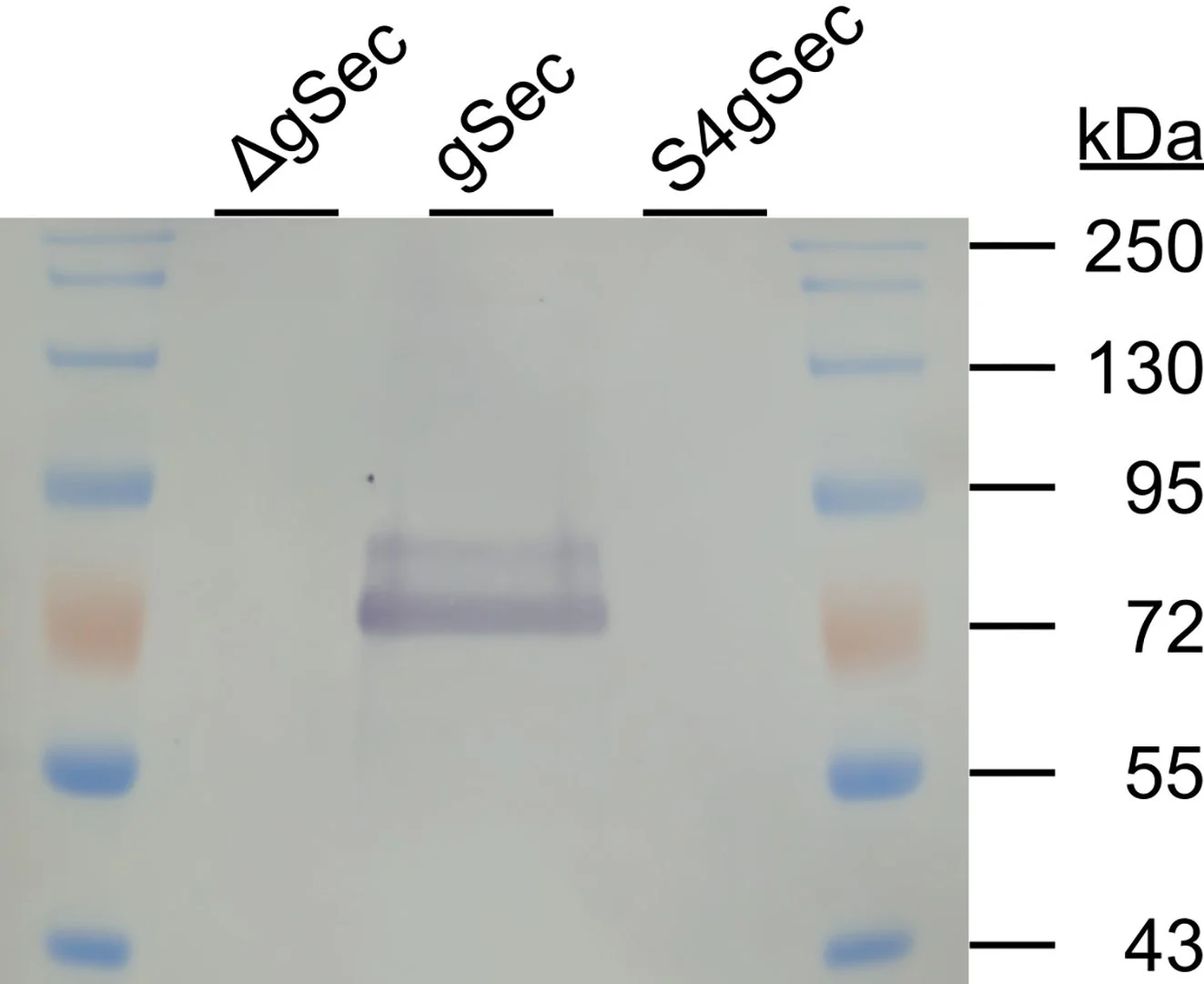
Anti-gliadin immunoblot of propan-2-ol extracted cell lysates of *K. phaffii* ΔgSec, gSec, and S4gSec. Electrophoresis was performed in a 10% tris-glycine gel, using the NEB prestained protein ladder 10–180 kDA.

**Figure 3:**
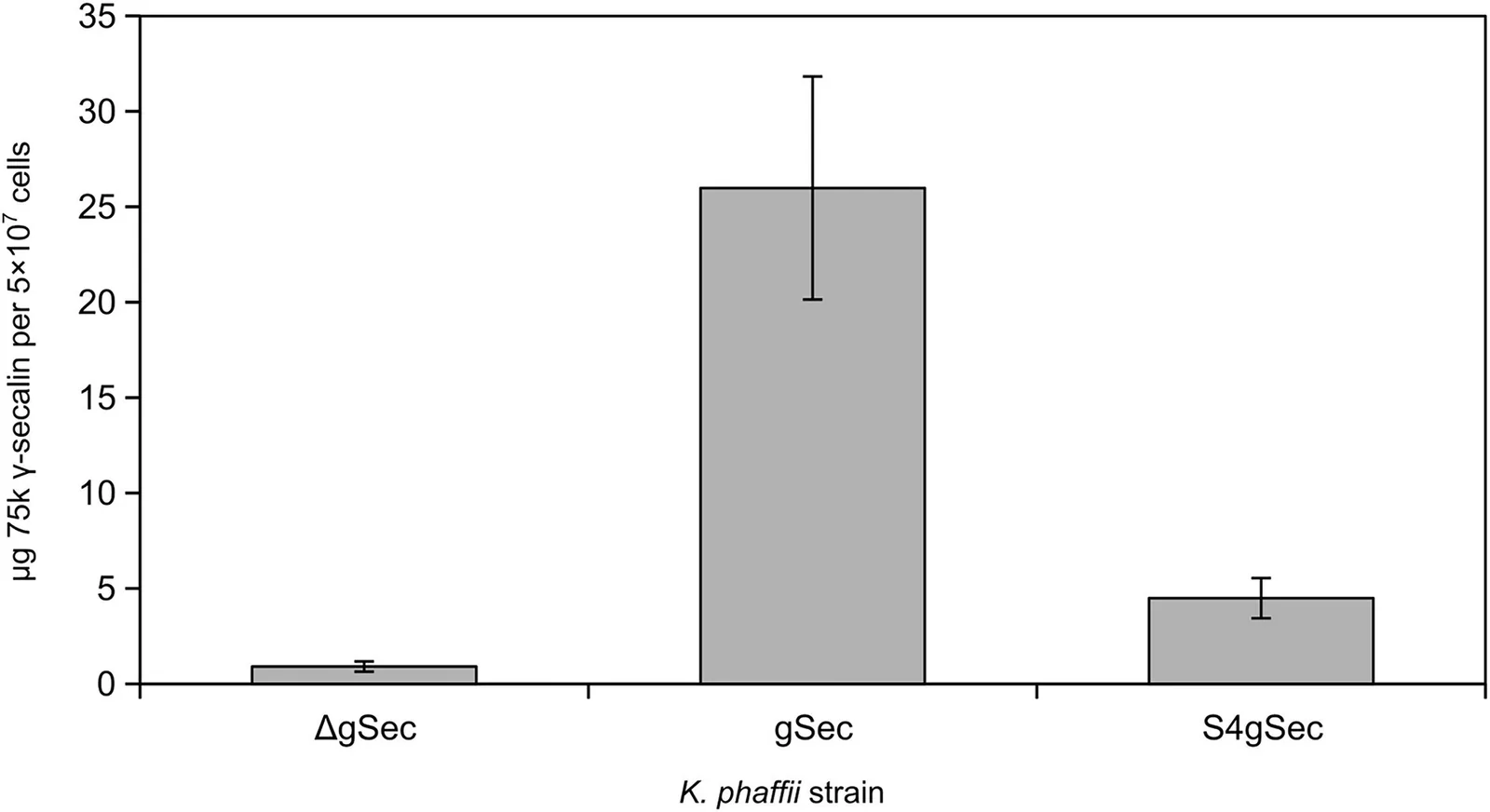
Competitive gluten ELISA with the R5 antibody. All strains were measured as propan-2-ol extracts in triplicate, results were normalized to 5 × 10^7^ cells. Both producing strains show significantly higher 75k γ-secalin yields (p_S4gSec_ = 7.7 × 10^–5^ and p_gSec_ = 0.018) compared to the null mutant *K. phaffii* ΔgSec. The null mutant shows a small signal due to measurement artifacts, amplified by the dilution factor.

Long read sequencing generated 2.23 million reads with a min Q score of 10 and an n50 of 10141 bases. Sniffles2 revealed reciprocal mates of the vector-chromosome junction as G]BSY3G-gSec:0] (SUPPORT = 36, VAF = 0.293) and a]chr4:676 361] (SUPPORT = 17, VAF = 0.207) for *K. phaffii* gSec. The junction for the null mutant vector ΔgSec was given as G]BSY3G-deltagSec:0] (SUPPORT = 96, VAF = 0.774) and a]chr4:676 361] (SUPPORT = 21, VAF = 0.223). The signal-sequence *OST1*-pre-MATα-pro mutant vector BSY3S4gSec showed a chromosome junction at G]BSY3S4G-gSec:4] (SUPPORT = 24, VAF = 0.960) and t]chr4:676 361] (SUPPORT = 6; VAF = 0.400). All reciprocal mates found with sniffles2 only mark one integration junction at the chromosome, therefore, the second junction was determined via IGV as chr4:676 362 for all three strains *K. phaffii* ΔgSec, gSec, and S4gSec. No read in any strain covered a vector-vector junction. Together with the well defined vector-chromosome junctions, this suggests the scarless replacement of the original expression cassette by integration of single copies of the different mutation cassettes in the respective strains.

*Komagataella phaffii* gSec, producing the native 75k γ-secalin, showed 31 differentially expressed genes (*P* ≤ 0.05) compared to the null-mutant *K. phaffii* ΔgSec, of which 22 are annotated. For five of the nine non-annotated genes, no protein family membership was predicted; Table 1 illustrates the putative proteins’ predicted properties. Six of the annotated genes were upregulated (log_2_ fold-change (log2(FC)) 0.23 to 0.54) and 16 annotated genes were downregulated (log2(FC) −0.16 to −0.64).

**Table 1:** Family membership and feature prediction of differentially expressed non-annotated genes in *K. phaffii* gSec. IDs were taken from PANTHER and InterPro respectively, features were predicted through InterPro.

Gene locus ID	log2/FC	Predicted family membership	ID	Predicted features
BQ9382_C2-1445	0.39	SAGA complex, Sgf11 subunit	IPR013246	Classic zinc finger
BQ9382_C2-1511	0.42	UPF0768 protein YBL029C-A	PTHR28139	Zinc ribbon 15
BQ9382_C2-1648	0.46	N/A	N/A	N/A
BQ9382_C2-5650	0.25	N/A	N/A	Consensus disorder prediction
BQ9382_C2-7005	0.53	N/A	N/A	N/A
BQ9382_C3-0780	0.48	N/A	N/A	Transmembrane protein
BQ9382_C3-2055	-0.33	N/A	N/A	N/A
BQ9382_C3-4340	0.33	N-acetylglucosamine-induced protein 1	PTHR35020	N/A
BQ9382_C4-1530	0.42	GDXG lipolytic enzyme	IPR050300	AB hydrolase fold

Gene ontology (GO) terms assign genes to biological processes, molecular functions, or cellular components (Ashburner et al. 2000), enabling the analysis of large amounts of biological data for overarching effects (Young et al. 2010). Enrichment analysis depends on the proportion of GO-terms within the set of differentially expressed genes compared to the proportion of the same terms in a reference dataset, here, *S. cerevisiae* S288C. The analysis of the upregulated genes did not result in statistically significant enrichments for *K. phaffii* gSec. GO-terms enriched within the downregulated gene set are shown in detail in Supplementary Table 1; generally, terms regarding DNA repair were more highly abundant.

In the strain comparison of *K. phaffii* S4gSec with *K. phaffii* ΔgSec, selection for a significance of *P* ≤ 0.05 yielded 830 differentially abundant mRNA sequences. Applying an absolute log_2_ fold-change criterion (|log2(FC)| ≥ 0.58) reduced the gene set to 349 candidates, comprising 207 annotated and 142 putative genes. Only the two putative genes at loci BQ9382_C2-7005 and BQ9382_C4-1530 were differentially expressed both in *K. phaffii* gSec and S4gSec (Fig. 4). A total of 29 annotated genes were downregulated; no GO-term was significantly enriched within this group. Among the 175 upregulated and annotated genes, 17 could not be mapped by PANTHER against *S. cerevisiae* genes, while four genes were multi-mapped. Detailed results of the GO-term enrichment analysis are shown in Supplementary Table 1; generally, enriched biological process terms comprised transmembrane transport and amino acid metabolism. Notably, the term “cellular response to topologically incorrect protein” was also enriched 5.75-fold through six significantly upregulated genes in *K. phaffii* S4gSec (shown as: *GENE* {log2(FC); P-adjusted}): *FES1* {0.71; 1.30E-02}, *HAC1* {0.63; 4.34E-02}, *KAR2* {0.83; 1.22E-02}, *OPI1* {0.79; 2.32E-02}, *TMC1* {0.60; 8.99E-04}, and *TRL1* {0.87; 1.22E-02}. Fes1p acts as a cytosolic nucleotide exchange factor for the HSP70 family ATPase *SSA1* (Kabani et al. 2002). The ATPase *KAR2*, also a HSP70 family member, mediates protein import into the ER (Dreier et al. 1995) and acts as a chaperone (Simons et al. 1995). Transcription factor Hac1^i^p, from *HAC1* mRNA unconventionally spliced in the cytosol, induces the unfolded protein response (Guerfal et al. 2010). *HAC1* translation and its activation through splicing is dependent on *TRL1*, a tRNA ligase (Mori et al. 2010). *OPI1*, a transcriptional regulator located within the ER membrane, controls the flux of lipid metabolites and is proposed to interact with UPR sensors through the ER membrane (Covino et al. 2018). The zinc finger protein Tmc1p is an effector involved in the proteotoxic stress response and, through its induction by Rpn4p, connected to proteasome abundance (Guerra-Moreno and Hanna 2016). *RPN4* itself, however, was not significantly affected in the *OST1*-pre-MATα-pro 75k γ-secalin producing mutant cells.

**Figure 4:**
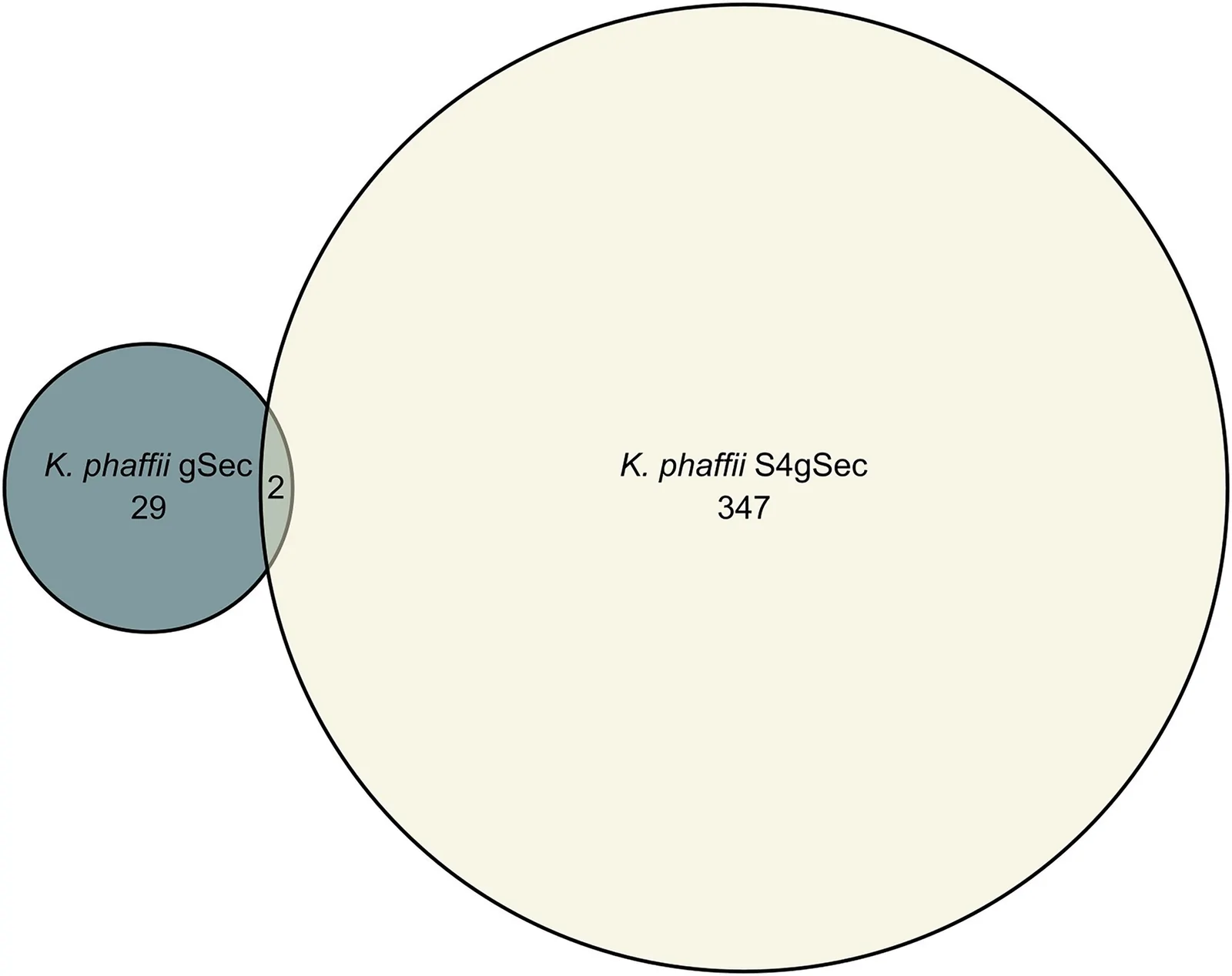
Number of differentially expressed genes in *K. phaffii* gSec and S4gSec, relative to *K. phaffii* ΔgSec. The absolute log_2_ fold-change criterion of *K. phaffii* S4gSec (|log2(FC)| ≥ 0.58) was not applied to *K. phaffii* gSec, since the effect size was too small in the significantly differentially expressed genes of this strain. Only the two upregulated putative genes at loci BQ9382_C2-7005 and BQ9382_C4-1530, are differentially expressed in both strains. A membership to the GDXG lipolytic enzyme family was predicted for the BQ9382_C4-1530 gene, no functional relationship was found *in silico* for BQ9382_C2-7005.

Cellular component analysis displayed enrichment of GO-terms associated with the peroxisome and the peroxisomal matrix. Annotated *K. phaffii* genes, which were not found under their gene name in the *S. cerevisiae* genome during the GO-term analysis, were further investigated through BLASTP in the Methylotroph Gene Order Browser (Douglass et al. 2019). This resulted in eleven alignments of *K. phaffii* proteins with the *S. cerevisiae* S288C proteome, while six gene products could not be mapped. High-scoring Segment Pairs (HSP) (Moreno-Hagelsieb and Latimer 2008) of these basic local alignment searches scored between 140 and 342, with higher scoring values indicating better fit of the local alignments. The HSP’s significance is given as E-value (expect value), where lower values suggest more significant matches. Table 2 displays the annotated *K. phaffii* genes with their *S. cerevisiae* matches.

**Table 2:** *K. phaffii* S4gSec genes differentially expressed compared to *K. phaffii* ΔgSec that were unmapped in GO-term enrichment analysis. Gene products were aligned to the *S. cerevisiae* S288C proteome with BLASTP, where higher HSP scores indicate longer stretches of high similarities between query and result and E-values measure the likelihood of random database entries generating an equal or higher score.

unmapped ID	gene locus ID	mapped to *S. cerevisiae* gene	HSP score	HSP E-value	cover in %	identity in %
*CYP559*	BQ9382_C1-1135	YHR007C (*ERG11*)	158	7E-11	38	26.54
*FMO6*	BQ9382_C1-4070	YHR176W (*FMO1*)	205	9E-17	56	27.15
*QOR2*	BQ9382_C1-6460	YNL134C	214	2E-18	78	26.43
*HXT400*	BQ9382_C1-8200	YDR536W (*STL1*)	299	3E-28	78	26.56
*ORT2*	BQ9382_C2-4100	YOR130C (*ORT1*)	202	2E-17	87	26.77
*SPS24*	BQ9382_C2-5675	YNL202W (*SPS19*)	147	2E-10	96	26.24
*QUP4*	BQ9382_C2-6990	YMR011W (*HXT2*)	342	7E-34	70	29.10
*PYX500*	BQ9382_C3-1730	YDR402C (*DIT2*)	140	1E-08	37	26.36
*FLO400*	BQ9382_C4-4530	YDR134C	81	2E-02	45	49.43
*CSD100*	BQ9382_C4-4560	YCL017C (*NFS1*)	144	2E-09	41	27.01
*FLO300*	BQ9382_C4-5335	HR211W (*FLO5*)	203	5E-15	9	25.63
* POX18*	BQ9382_C1-2160	N/A				
* PMP4*	BQ9382_C1-3445	N/A				
* LRA41*	BQ9382_C1-3465	N/A				
*CAO1*	BQ9382_C1-6490	N/A				
*ATG30*	BQ9382_C3-1735	N/A				
*AYF1*	BQ9382_C3-6390	N/A				

Three of the six annotated *K. phaffii* FLO-genes, which are involved in flocculation, were significantly upregulated in the S4gSec strain: *FLO300* {3.37; 2.68E-83}, *FLO5-1* {1.41; 3.92E-05}, and *FLO400* {1.15; 6.21E-04}). Additionally, the non-annotated upregulated genes BQ9382_C3-6507 {4.35; 2.79E-131} and BQ9382_C4-5308 {1.41; 6.41E-05} were predicted to show flocculin type 3 repeats (Pfam entry PF13928). Transcription factors acting upon FLO-genes, *GCN4* (Hinnebusch and Natarajan 2002) {1.18; 2.55E-05} and negative *FLO5-1* regulator *NRG1* (De et al. 2025) {-0.86; 3.70E-05}, are significantly affected in the *K. phaffii* S4gSec strain. Apart from the two proteins with predicted flocculin repeats, 13 non-annotated, differentially expressed genes were predicted to be transmembrane proteins. Two of the gene products were predicted to be membrane-bound, and one (BQ9382_C1-5451) showed a 100% homology to the major facilitator superfamily (MFS) profile domain-containing protein XP_002 490 371 described in the *K. phaffii* GS115 wildtype (De Schutter et al. 2009). Genes of the cell wall integrity and stress response component (WSC) family, indicated in sensing extracellular methanol in the environment (Ohsawa et al. 2022), are not significantly differentially expressed. Only one of the known transcription factors that induce the methanol metabolism, *MXR1* (Lin-Cereghino et al. 2006), was overexpressed in *K. phaffii* S4gSec.

## Discussion

*Komagataella phaffii* gSec produced the expected amounts of 75k γ-secalin. ELISA of the null mutant whole cell extracts indicated trace amounts, which can be attributed to measurement artifacts. The strain harbouring the native *Sec2*-gene intracellularly accumulated 639.97 ± 155.93 mg/l 75k γ-secalin, surpassing the published gluten protein yields of heterologous systems such as *E. coli* (50 mg/l (Dowd and Bekes 2002)) and *S. cerevisiae* [10 mg/l (Pratt et al. 1991)]. Despite the lack of target protein secretion, otherwise a key feature of the *K. phaffii* expression system, this demonstrates the yeast’s general utility for heterologous gluten protein production. The goal of further system optimization towards secretory expression for easier industrial applicability lead to introduction of different secretion signal peptides. As reported earlier (Büchner et al. 2025), the strain producing the *OST1*-pre-MATα-pro signal-sequence mutant showed no bands in the immunoblot; however, a small but significant signal is detectable in the ELISA measurement. This may occur due to fragmentation of the target protein which would leave peptides too small to form bands in SDS-gels, but still large enough to elicit ELISA signals. The gene expression data described here can aid in uncovering the underlying mechanism through further studies.

All strains showed seamless integration of the singular expression cassettes between 676 361 bp and 676 362 bp of chromosome 4 in reverse orientation. This position occurred through the random integration of the original expression cassette, which was later replaced by predominantly homologous vectors carrying mutated versions of the target gene and a different antibiotic resistance marker (Büchner et al. 2025). The identical integration site in all strains points to a successful replacement of the original cassette through homologous recombination, even though *K. phaffii* shows a relatively low gene targeting efficiency (Ito et al. 2018). The site itself is not annotated as a coding sequence, but lies in the intergenic region of *STL1* (end at 675 945 bp) and *PHO86* (start at 676 659 bp). This potentially influences the expression of the ER-resident phosphate transporter related Pho86p, which is required for the function of phosphate transporter Pho84p (Lau et al. 2000). Within the 714 bp sequence upstream of *PHO86*, no transcription factor sequences were predicted (Monteiro et al. 2019). It is, however, possible that the site harbours some yet unknown transcription factor binding sites that could be disrupted by the vector integration. Instead of being compared to the *K. phaffii* BSY BG11 wild type, *K. phaffii* gSec and S4gSec were compared to null mutant ΔgSec, which contains the same chromosome-vector-junctions as the two 75k γ-secalin production strains. Therefore, possible effects of the integration site on the cell metabolism are expected to be similar for the whole panel of analyzed strains and are not part of this transcriptome analysis. Phenotypically, the investigated strains did not exhibit the slowed growth characteristics consistent with phosphate starvation, which would be expected from strains with downregulated *PHO86* expression (Subitha et al. 2020).

GO-term analysis of downregulated genes in *K. phaffii* gSec (Supplementary Table 1) revealed an enrichment of terms related to DNA repair and maintenance, as well as meiotic and mitotic processes. Downregulation of such crucial cellular functions might indicate a lessened vitality. Although the log_2_ fold changes of the downregulated genes are significant (*P* ≤ 0.05), the effect sizes are small, with log2(FC) ranging from −0.16 to −0.63, and no significant impact on cell growth behaviour is observed. In general, only about 0.6% of the 5385 mRNAs given in the *K. phaffii* reference genome were significantly differentially expressed in the native 75k γ-secalin producer *K. phaffii* gSec. On the one hand, this supports and expands on previous findings that expression of the target protein did not affect ER homeostasis in this system. On the other hand, the small number of 31 differently expressed genes, including 29% non-annotated genes, may render further analyses, such as GO-term enrichment, prone to effect overestimation.

*Komagataella phaffii* S4gSec analysis showed an enrichment of terms regarding cellular response to topologically incorrect protein, implying challenges for cell homeostasis through *OST1*-pre-MATα-pro 75k γ-secalin expression. In general, when confronted with adverse environmental conditions, *K. phaffii* responds by upregulating the flocculin (FLO) gene family through transcriptional regulator Flo8p (Kobayashi et al. 1996), inducing pseudo-hyphal growth (De et al. 2020). Despite the up-regulation of three known FLO-genes and two non-annotated genes with flocculin type three domains in *K. phaffii* S4gSec, *FLO8* did not show significant differential expression. Instead, the transcription factor *GCN4*, involved in *S. cerevisiae* flocculation (Braus et al. 2003), is upregulated in *K. phaffii* S4gSec, while the negative *FLO5-1* regulator *NRG1* (De et al. 2025) is downregulated. The latter, a zinc finger transcriptional repressor, is expected to be downregulated in the absence of glycerol or glucose (Wang et al. 2016). However, since all samples in this study were taken from cultures growing on methanol as the sole carbon source, the gene would be expected to be equally downregulated in the expression strains and in the reference null mutant. Therefore, other effects than glycerol or glucose depletion may cause the further relative downregulation of *NRG1* in *K. phaffii* S4gSec: *NRG1* regulation through *SKN7* [(MacIsaac et al. 2006) as per Saccharomyces Genome Database (Wong et al. 2023), section “NRG1/YDR043C Regulation, Regulators, High-throughput”] suggests an effect related to oxygen stress. This is supported by the GO-term enrichment of peroxisome-related genes, which are coincidentally also affected by the *GCN4* master regulator (Natarajan et al. 2001). On a phenotypic level, neither pseudohyphal growth nor flocculation were observed, the latter may be due to a lack of calcium needed to form aggregates in the complex media (Stratford 1989).

Gene ontology enrichment of histidine metabolism genes concords with the upregulation of *GCN4*, which, in *S. cerevisiae*, acts as a transcriptional activator for the genes *HIS1, HIS4*, and *HIS7*, which were upregulated in this study (as per Saccharomyces Genome Database (Wong et al. 2023), section “GCN4/YEL009C Regulation, Targets, High-throughput”). The *GCN4* transcription factor stimulates the transcription of genes involved in 12 different amino acid biosynthetic pathways in response to amino acid starvation; a regulatory response known as general amino acid control (GAAC) (Hinnebusch and Natarajan 2002). Furthermore, *GCN4* was shown in *S. cerevisiae* to engage in crosstalk with the ER-stress transcription factor *HAC1*, and both factors acting on the same promoter regions of the *FLO11* flocculin (Herzog et al. 2013). However, *FLO11* showed no significant difference in abundance in this study. The only other transcription factor indicated in the regulation of all three upregulated HIS-genes in yeast is *S. cerevisiae ZNF1* (Wong et al. 2023). Protein blast of the *S. cerevisiae ZNF1* sequence revealed 9% homology to Pp*TRM1* (HSP 42.4, E-value 1E-04), a gene not significantly differentially expressed in *K. phaffii* S4gSec and thereby excluded as an effector in the histidine metabolism within the parameters of this study.

Nevertheless, the upregulation of the *GCN4* transcription factor, combined with the enrichment of the GO-term “ammonium transmembrane transport”, points to an amino acid stress-induced transcription. Gcn4p also plays an important role in the Hac1^i^p induced UPR, since it is required for the induction of a majority of UPR responsive genes (Patil et al. 2004, Herzog et al. 2013).

ER-stress transcription factor *HAC1* mRNA has a half-life of approximately 4.8 min under relaxed conditions and 9.8 min under ER-stress conditions (Sarkar et al. 2018). Its activation relies on complex processing and degradation mechanisms (Cherry et al. 2019) and results in a protein that is quickly degraded (Pal et al. 2007). Therefore, a time series study targeting the relative expression of stress-related transcription factors *HAC1* and *GCN4* might elucidate further mechanistic interrelation in *K. phaffii* S4gSec relative to *K. phaffii* ΔgSec. In general, *GCN4* responsive elements are known for *S. cerevisiae* (Mösch et al. 1990), but are not explicitly described as structural elements in *K. phaffii*. The panel of upregulated genes presented here enables further studies in *K. phaffii* promoter elements through e.g. fluorescence reporters, specifically regarding genes affected by *GCN4* regulation. Although stress through amino acid depletion in the *K. phaffii* S4gSec cultures is a possible cause for GAAC upregulation and heightened FLO-gene mRNA abundance hints at extracellular stressors, the similar growth behaviour of all strains does not suggest starvation or other environmental stress as root causes for *K. phaffii* S4gSec’s aberrant gene transcription behaviour.

The heightened transcriptional response to the signal sequence mutant protein, together with the target protein detection only in the ELISA of the extracted *K. phaffii* S4gSec whole cell lysates but not in the immunoblot, hints at the protein being synthesized and then being degraded. The exact route of translated 75k γ-secalin in *K. phaffii* cells is unknown so far. The base-level UPR of the organism may also hinder the detection of any UPR-related degradation, since it may not require additional upregulation of the pathway. The only indication of ER-localized protein challenge in *K. phaffii* S4gSec is the upregulation of *FES1, KAR2, OPI1, TRL1*, and *TMC1*. While *FES1* takes on a central role in the ubiquitin-dependent degradation of misfolded proteins (Gowda et al. 2013), its activating factor *HSF1* is downregulated in the secretion signal mutant (Ciccarelli et al. 2023). Contrary to its canonical role as a gene repressor, *OPI1* induces *e.g*. histidine biosynthesis (Panessa et al. 2023), suggesting a connection to the significant enrichment of the GO-term “L-histidine biosynthetic process” in the upregulated genes (Supplemental Table 1). Furthermore, *OPI1* responds to inositol depletion, linking it to UPR upregulation (Hancock et al. 2006). The ER resident BiP chaperone *KAR2* is a central trigger for the UPR, activating it by dissociating from the sensory molecule Ire1p, which in turn splices the mRNA of the transcription factor *HAC1* (Okamura et al. 2000). The ligase *TRL1* joins cleaved pre-RNA exon halves and thereby plays a central role in non-conventional *HAC1* mRNA splicing and UPR induction (Köhler et al. 2025). Quantification of *HAC1* abundance in this study did not differentiate between the induced and uninduced mRNA variant, and does therefore in itself not allow any conclusions about the stress-state of the ER. *TMC1* on the other hand is an effector of the non-UPR related *RPN4* proteotoxic stress response, that mainly regulates the abundance of proteasomes (Guerra-Moreno and Hanna 2016). The log2(FC) of the genes lies between 0.60 (*TMC1*) and 0.87 (*TRL1*), indicating a moderate protein-based challenge to the ER. Together with the tendency of gluten proteins to accumulate in the ER (Rosenberg et al. 1993), the detection pattern observed in *K. phaffii* S4gSec ELISAs and immunoblots might stem from ER associated gluten protein degradation. However, deeper research into the specific mechanisms behind the secretion signal related degradation is needed, e.g. through time-resolved experiments, co-immunoprecipitation, and separation of cell lysates through density gradient centrifugation and extraction of gluten from the fractions.

The general amount of genes significantly affected in the strain expressing *OST1*-pre-MATα-pro 75k γ-secalin is 26.8-fold higher than in *K. phaffii* gSec. This hints at the metabolic impact of the protein secretion leader, but the data must be regarded cautiously, as the gene dosage influence on phenotype may vary between genes (Rentzsch et al. 2025) and is not known for many of the described genes in this system.

Furthermore, only around 59.3% of the 349 significantly differentially expressed genes in *K. phaffii* S4gSec are currently annotated. As with the differential expression data from *K. phaffii* gSec, where 29% of the 31 differentially expressed genes were non-annotated, the share of genes without verified function renders transcriptome data interpretation speculative, as GO-term enrichment analysis relies on the proportion of differentially expressed known genes in relation to a reference transcriptome (Young et al. 2010). The high proportion of unknown genes makes direct experimental confirmation of the transcriptome study’s results challenging, since it calls for extensive investigations of the affected genes, e.g. through fluorescence reporters. In addition to the non-annotated genes, the annotated, differentially expressed genes of the *K. phaffii* S4gSec include six genes that could not be mapped to *S. cerevisiae*. Together with the amount of non-annotated genes differentially expressed in this strain, the data highlight the need for further characterization of the *K. phaffii* system beyond its similarities to more widely researched model organisms. This could enable a more nuanced interpretation of the data, e.g. through the application of bias-reducing analysis methods such as Gene Set Enrichment Analysis.

## Conclusion

With a yield 639.97 ± 155.93 mg/l native 75k γ-secalin, the *K. phaffii* production system surpasses the published gluten protein yields of other heterologous systems. An attempted production optimization through exchange of the secretion leader peptide had a strongly adverse effect on the yield. Therefore, this study investigated the influence of native 75k γ-secalin expression and expression of the signal-sequence mutant *OST1*-pre-MATα-pro 75k γ-secalin on the transcriptome of *K. phaffii*. The results indicate a moderate transcriptional response to the production of the native rye prolamin in strain *K. phaffii* gSec, while the signal-sequence mutant strain *K. phaffii* S4gSec displays broader transcriptional changes. In the latter, stress-related mechanisms such as flocculation, the unfolded protein response, and general amino acid control (GAAC) were upregulated, revealing the transcription factor *GCN4* as a noteworthy target for further expression studies. Stress-related gene expression notwithstanding, the strains’ growth behaviour remained unaltered, thereby indicating intracellular causes of the observed transcriptome changes. The differences in the transcriptomes of *K. phaffii* strains gSec and S4gSec may highlight the substantial impact of secretion-leader characteristics. However, the high proportion of non-annotated genes in both datasets, as well as the limited knowledge of *K. phaffii* gene regulatory elements, underscores current limitations in the biological interpretation of the data and their experimental verification. Continued functional characterization of the *K. phaffii* genome through reporter strains, paired with complementary approaches such as time-resolved expression profiling and gene-set enrichment analysis, will help refine mechanistic insights into protein-production dynamics and the impact of secretion signals on this system.

## Supplementary Material

xtag046_Supplemental_Files

## Data Availability

The authors confirm that the data supporting the findings of this study are available within the article and its Supplementary materials. Raw data of the paired end reads are openly available in zenodo.org at https://zenodo.org/records/17726183, DOI 10.5281/zenodo.17726182. Raw data of the long reads are not publicly available due to the partial sequences of the proprietary pBSY3Z and pBSY3S4Z plasmids contained within.
