# Supplementary material for: A chimeric rather than native secretion signal elicits stress-related transcriptional adaption during 75k γ-secalin production in Komagataella phaffii
Source: FEMS Microbes. 2026 Oct 7;7:xtag046. doi: 10.1093/femsmc/xtag046 (PMC13656322; doi:10.1093/femsmc/xtag046)
Supplement: Supplementary file 1 [file xtag046_supplemental_files.zip › FEMSMC-2026-006.R2 supplementary data - reviews and author response.docx]

**Reviews of manuscript FEMSMC-2026-006**

**Reviewer 1: Ravinder Kumar, UCSF**

**Recommendation: Major Revision**

My comments to authors are below

1) Mention which plasmid was used

2) Method of yeast transformation need to be included

3) Whether plasmid was linearized , if yes which enzyme was used

4) Site of integration need to mention

5) Show protein expression with western blot and SDS-PAGE

**Reviewer 2**

**Recommendation: Reject**

Authors should carried out experiments to confirm the conclusions.Therefore, I must recommed to reject this manuscript.

**Associate Editor Recommendation: Major Revision**

**EIC Decision: Major Revision**

**Author's Response to Decision Letter for (FEMSMC-2026-006)**

The authors thank the reviewers for their time and their constructive comments. The input helped to substantiate the manuscript.

- We expanded on the strain construction in a concise manner to better illustrate the vector at hand and the cloning strategy.

- The integration site of the cassette is a valid point as it is potentially influential on the behaviour of the strain. To obtain the most information possible on this, we conducted a long read sequencing experiment that showed seamless integration of the cassette into the intergenic region of chromosome 4 for all three strains. Potential consequences for the strains are discussed briefly.

- 75k γ-secalin production was verified by immunoblot and ELISA-measurements, since the S4gSec strain does show a signal in the ELISA, but no bands in the blot. This is discussed briefly. K. phaffii gSec showed both, blotting and ELISA signals. All strains were consistent with their behaviour reported earlier.

- We agree that direct experimental validation would strengthen the conclusions; however, coupling a fluorescence reporter to a GCN4 affected promoter is not currently tractable, as the relevant binding sites and promoter regions for this transcription factor have not been characterised. De novo identification would require substantial additional resources and time that fall outside the scope of the present study. We have instead clarified the exploratory nature of the paper, and we have revised the manuscript to state these limitations explicitly and to frame our conclusions accordingly in the discussion.

**Reviews of manuscript FEMSMC-2026-006.R1**

**Reviewer 1: Ravinder Kumar, UCSF**

**Recommendation: Accept**

**Reviewer 3**

**Recommendation: Minor Revision**

The manuscript by Büchner et al. describes the transcriptomic response of K. phaffii strains expressing secalin, an aggregation-prone protein, with the help of two different secretion leader sequences.

It is already a revised version (which was reviewed by others before), and the new data and explanations clearly add to better understand the research. However, there are some remaining points that need to be addressed:

For me the most puzzling question is where the secalin with hybrid OST1-pre-MATα-pro secretion leader is within the cell? It must be somewhere, however cannot be detected by Western Blotting of the cell lysates. Do the authors have any idea which cellular parts (organelles) are present within their cell lysates? As there is a distinct transcriptomic response, the secalin is most likely synthesized/translated and probably also enters the secretory pathway. Its fate afterwards is unknow so far, it can be stuck during translocation or being degraded. This should also be discussed in a bit more detail.

• Please set all genes names to italics e.g. GCN4 in line 20.

• Line 45: …ER import …

• Line 46: …bipartite S. cerevisiae mating factor α (MFα) secretion leader.

• Line 65: What is meant with “null mutant”? The non-producing wild type? Or a mock strain/empty vector control (EVC), that carries the empty expression plasmid?

• Line 65ff: …differences between the non-producing control strain, and the

• strains producing secalin with either its native signal sequence, or the hybrid OST1-pre-MATα-pro secretion leader.

• Line 76: electroporation or elctro-transformation (only mammalian cells get transfected)

• Line 89 and elsewhere: please replace “fermentation” by “cultivation”, as you have aerobic and not fermentative cultures

• Line 103: …EDTA), the pellets…

• Line 167ff: I find the start of the Results section rather abrupt. It would be good to have at least one sentence defining the experimental goals before starting to describe the results.

• Line 171ff: What about secreted secalin in the supernatant? Was this also determined?

• Table 2: the lower part seems to be missing.

• It would be helpful to include the figure of a Venn diagram of the differentially expressed genes.

• The title of the paper/header in Suppl. File 1 needs to be updated.

• Line 226: I was not aware that GCN4, the activator of amino acid biosynthesis, was directly controlling FLO gene expression. This is discussed in more detail in the discussion section., however, the connection between HAC1 and GCN4 is not mentioned. Please add this to the discussion section.

• Line 246: Please state if the integration site “676361 bp and 676362 bp of chromosome 4“ was intended (by a homologous fragment on the vector) or occurred randomly.

• Line 254: please replace “evinces” with a more suitable word e.g. “contains” or “shows”

• Results: it would be helpful to explain a few more words regarding the mentioned differentially expressed genes.

• Line 287: “peroxysome“ should be „peroxisome”. Please correct in the whole manuscript.

• Line 320ff: was the gene copy number of secalin determined in the two expression strains? Or can it be deduced from the nanopore sequencing by sequence coverage?

• Line 329ff: I do not fully agree with the newly added sentence. Promoter sequences are usually upstream of the start codon, and can be easily deduced from the genome, even for genes without known function. However, I agree that the large number of differentially expressed genes without function makes data interpretation more complicated, as it would be necessary to either overexpress or knockout the uncharacterized genes to deduce their function.

• Is intracellular secalin an industrially relevant approach?

**Associate Editor Recommendation: Accept**

**EIC Decision: Minor Revision**

**Author's Response to Decision Letter for (FEMSMC-2026-006.R1)**

We would like to thank the reviewers for their time, effort, and the thorough analysis of our paper. The remarks and questions raised helped us to interpret the experimental results more clearly and to identify knowledge gaps more precisely in the text. The suggestions were incorporated and all adjustments were marked in the manuscript. In this response, only the more complex remarks are addressed; due to the shifts in line numbers, we inserted the new and reworked sentences in this letter.

• For me the most puzzling question is where the secalin with hybrid OST1-pre-MATα-pro secretion leader is within the cell?

This question is indeed puzzling and we can currently give no clear answer. However, we included a section arguing for the possible ER related degradation and discuss possible strategies for further investigation:

“The heightened transcriptional response to the signal sequence mutant protein, together with the target protein detection only in the ELISA of the extracted whole cell lysates but not in the immunoblot, hints at the protein being synthesised and then being degraded. The exact route of translated 75k γ-secalin in K. phaffii cells is unknown so far. The base-level UPR of the organism may also hinder the detection of any UPR-related degradation, since it may not require additional upregulation of the pathway. The only indication of ER-localised protein challenge in K. phaffii S4gSec is the upregulation of FES1, KAR2, OPI1, TRL1, and TMC1. While FES1 takes on a central role in the ubiquitin-dependent degradation of misfolded proteins (Gowda et al. 2013), its activating factor HSF1 is downregulated in the secretion signal mutant (Ciccarelli et al. 2023). Contrary to its canonical role as a gene repressor, OPI1 induces e.g. histidine biosynthesis (Panessa et al. 2023), suggesting a connection to the significant enrichment of the GO-term “L-histidine biosynthetic process” in the upregulated genes (Supplemental Table 1). Furthermore, OPI1 responds to inositol depletion, linking it to UPR upregulation (Hancock, Behta, and Lopes 2006). The ER resident BiP chaperone KAR2 is a central trigger for the UPR, activating it by dissociating from the sensory molecule Ire1p, which in turn splices the mRNA of the transcription factor HAC1 (Okamura et al. 2000). The ligase TRL1 joins cleaved pre-RNA exon halves and thereby plays a central role in non-conventional HAC1 mRNA splicing and UPR induction (Köhler et al. 2025). Quantification of HAC1 abundance in this study did not differentiate between the induced and uninduced mRNA variant, and does therefore in itself not allow any conclusions about the stress-state of the ER. TMC1 on the other hand is an effector of the non-UPR related RPN4 proteotoxic stress response, that mainly regulates the abundance of proteasomes (Guerra-Moreno and Hanna 2016). The log2(FC) of the genes lies between 0.60 (TMC1) 0.87 (TRL1), indicating a moderate protein-based challenge to the ER. Together with the tendency of gluten proteins to accumulate in the ER (Rosenberg et al. 1993), the detection pattern observed in K. phaffii S4gSec ELISAs and immunoblots might stem from ER associated gluten protein degradation. However, deeper research into the specific mechanisms behind the secretion signal related degradation is needed, e.g. through time-resolved experiments, co-immunoprecipitation, and separation of cell lysates through density gradient centrifugation and extraction of gluten from the fractions.”

• Line 46: …bipartite S. cerevisiae mating factor α (MFα) secretion leader.

Both abbreviations, Mfα and MATα are found in the literature. We opted to leave the MATα designation in keeping with the preceding publication, so that the strain names are consistent.

• Line 65: What is meant with “null mutant”? The non-producing wild type? Or a mock strain/empty vector control (EVC), that carries the empty expression plasmid?

• Line 65ff: …differences between the non-producing control strain, and the strains producing secalin with either its native signal sequence, or the hybrid OST1-pre-MATα-pro secretion leader.

“...differences between the non-producing translation-null mutant as the control strain, and strains producing secalin with either its native signal sequence, or the hybrid OST1-pre-MATα-pro secretion leader.”

• Line 167ff: I find the start of the Results section rather abrupt. It would be good to have at least one sentence defining the experimental goals before starting to describe the results.

“Previously published research regarding the optimisation of 75k γ-secalin production in K. phaffii showed a significant impact of the secretion leader signal on the protein yield (Büchner K et al. 2025). This study aims at elucidating the underlying mechanisms on a transcriptional level to provide starting points for future optimisation approaches and their monitoring. In order to track any phenotypic effects of the 75k γ-secalin expression, cell growth was measured during the 96 h methanol fed-batch phase in the shaker flasks.”

• Line 171ff: What about secreted secalin in the supernatant? Was this also determined?

“Also in accordance with these previously published results, neither immunoblot nor ELISA detected any 75k γ-secalin in the culture supernatant.”

• Table 2: the lower part seems to be missing.

Some genes could not be identified through comparison with S. cerevisiae. These were now more clearly designated as “N/A”.

• It would be helpful to include the figure of a Venn diagram of the differentially expressed genes.

A Venn diagram was included as Figure 4.

• Line 226: I was not aware that GCN4, the activator of amino acid biosynthesis, was directly controlling FLO gene expression. This is discussed in more detail in the discussion section., however, the connection between HAC1 and GCN4 is not mentioned. Please add this to the discussion section.

“GCN4 was shown in S. cerevisiae to engage in crosstalk with the ER-stress transcription factor HAC1, and both factors acting on the same promoter regions of the FLO11 flocculin (Herzog et al. 2013).”

“Gcn4p also plays an important role in the Hac1ip induced UPR, since it is required for the induction of a majority of UPR responsive genes (Herzog et al. 2013, Patil, Li, and Walter 2004).”

• Line 246: Please state if the integration site “676361 bp and 676362 bp of chromosome 4“ was intended (by a homologous fragment on the vector) or occurred randomly.

“This position occurred through the random integration of the original expression cassette, which was later replaced by predominantly homologous vectors carrying mutated versions of the target gene (Büchner K et al. 2025). The identical integration site in all strains points to a successful replacement of the original cassette through homologous recombination, even though K. phaffii shows a relatively low gene targeting efficiency (Ito et al. 2018).”

• Results: it would be helpful to explain a few more words regarding the mentioned differentially expressed genes.

“Fes1p acts as a cytosolic nucleotide exchange factor for the HSP70 family ATPase SSA1 (Kabani, Beckerich, and Brodsky 2002). The ATPase KAR2, also a HSP70 family member, mediates protein import into the ER (Dreier, Hartmann, and Rapoport 1995) and acts as a chaperone (Simons et al. 1995). Transcription factor Hac1ip, from HAC1 mRNA unconventionally spliced in the cytosol, induces the unfolded protein response (Guerfal et al. 2010). HAC1 translation and its activation through splicing is dependent on TRL1, a tRNA ligase (Mori et al. 2010). OPI1, a transcriptional regulator located within the ER membrane, controls the flux of lipid metabolites and is proposed to interact with UPR sensors through the membrane (Covino, Hummer, and Ernst 2018). The zinc finger protein Tmc1p is an effector involved in the proteotoxic stress response and, through its induction by Rpn4p, connected to proteasome abundance (Guerra-Moreno and Hanna 2016). RPN4, however, was not significantly affected in the 75k γ-secalin producing mutants.”

The genes explicitly mentioned in this section were then incorporated into the discussion of the protein localisation.

• Line 320ff: was the gene copy number of secalin determined in the two expression strains? Or can it be deduced from the nanopore sequencing by sequence coverage?

The singular integration was deduced form the sequence coverage:

“No read in any strain covered a vector-vector junction. Together with the well defined vector-chromosome junctions, this suggests the integration of single copies of the different expression cassettes in the respective strains.”

Again, we would like to thank Reviewer 3 for the helpful suggestions and hope to have them addressed in a sufficient manner.

Kai Büchner

On behalf of all authors

**Reviews of manuscript FEMSMC-2026-006.R2**

**Reviewer 1: Ravinder Kumar, UCSF**

**Recommendation: Accept**

Author have responded to all quires, draft is good enough for acceptance in its present form.

**Reviewer 3**

**Recommendation: Accept**

The authors have answered all my questions and made the respective modifications to the manuscript.

**Associate Editor Recommendation: Accept**

**EIC Decision: Accept**
